# Join our team, change the world: edibility, producibility and food futures in cultured meat company recruitment videos

**DOI:** 10.1080/15528014.2021.1884787

**Published:** 2021-03-31

**Authors:** Neil Stephens

**Affiliations:** Social and Political Sciences, Brunel University London, Uxbridge, UK

**Keywords:** Cultured meat, edibility, producibility, sociology of expectations, recruitment videos, cell-based meat, clean meat, cultivated meat, Memphis Meats

## Abstract

Cultured meat is a novel technology that uses tissue engineering to expand cells taken from animals to grow muscle for consumption as food. Those supporting the technology anticipate it could radically disrupt livestock farming with, they propose, significant benefits for the environment, human health, and animal wellbeing. This paper examines the emergence of this sector through the prism of one of the leading companies – Memphis Meats – in particular focusing upon their online recruitment activity in online videos. Founded in 2015, by 2020 they had announced investment of over $160 m to build a pilot-plant and recruit staff to bring cultured meat closer to commercialization. This paper argues the company’s recruitment videos work to enact what I term “producibility”, a concept aligned to existing work on “edibility”, that emphasizes the process of becoming that foodstuff (included novel foodstuffs) undergo. I deploy existing theoretical work on multiple categories of futures – big/little, individual/institutional/field – to analyze Memphis Meats’ online recruitment activity. I argue that, by entangling science and food futures, the company’s videos work to assert the status and politics of cultured meat, render it producible and edible, and articulate a novel and transformative food-professional identity: the cultured meat producer.

## Introduction

Cultured meat, also known as cell-based meat, clean meat, and cultivated meat (CM), is a technology that uses the techniques of tissue engineering to produce animal muscle for consumption as food (Langelaan et al. 2010; Stephens et al. 2018). Those developing the technology stress the potential environmental, animal welfare, and health benefits compared to livestock meat (Datar and Betti 2010). Research in the field now has a near twenty-year history. The first wave of researchers was largely university-based and struggled to attract sustained funds for their research (Stephens, Sexton, and Driessen 2019). The defining moment of the field was in 2013, when Professor Mark Post of Maastricht University produced the world’s first burger grown in a laboratory, which was subject to a taste test in a live press conference-come-cookery show in London (O’Riordan, Fotopoulou, and Stephens 2017; Post 2014). The burger tasting had been funded by Google co-founder Sergi Brin, who had anonymously responded to Post’s claim in the press that meat produced this way was possible. Following the burger the field entered a second wave of activity, this time largely based on a start-up culture driven by venture capital seed funds. This new source of funding was often mission-based (driven by social benefits as well as profit), less risk obverse, and, like the funding for the 2013 burger, often linked to Silicon Valley’s abundant IT finances. As such, CM occupies part of an emergent food tech imaginary that variously also includes plant-based meats (Sexton 2016), novel presentations of insects (House 2018), and 3D-printed foods (Lupton and Turner 2018).

The first start-up to enter the CM field was Modern Meadow in 2011, a company founded by the father and son team who had previously successfully launched the first 3D bioprinting company Organovo four years earlier. Modern Meadow produced some early demonstration products – a small morsel of meat and dried steak chips – but then decided to focus the company entirely on producing cultured leather. The next company to get established, Memphis Meats, is the core focus of this paper. Since foundation in 2016, Memphis Meats have released videos of their prototype CM foods, including a beef meatball, chicken, and duck. However, at the time of writing, they nor any other CM company, have commercially released any products. Also like other CM companies, Memphis Meats invoke a vision of the future in which their product leads to social benefits. As the front page of their website claims, “We’re making meat that is better for animals and that at scale uses significantly less land, water, energy and food inputs. Our process will produce less waste and dramatically fewer greenhouse gas emissions. We believe that the planet will be the ultimate beneficiary of our product.” (Memphis Meats 2018).

Since Memphis Meats was established, a number of other companies have entered the space. Many, like Memphis Meats, are based in the San Francisco Bay area, such as Mission Barns, Wild Type, and Finless Foods (who make cultured fish). Another San Francisco-based company is Eat Just (formerly Hampton Creek) who, unlike the others, already was a successful business producing vegan products including mayonnaise. Mark Post’s Maastricht group also formed an allied start-up, Mosa Meats, and these three, Mosa Meats, Memphis Meats, and Just, are perhaps seen as the most visible and leading companies. Some estimates suggest as many as sixty companies exist globally at various stages of development. This given, Memphis Meats was the first to raise Series A funds, and is seen as an industry leader.

In this paper, I analyze a particular aspect of Memphis Meats’ work: their deployment of YouTube videos to promote both CM and their company, and most specifically here, their release of a set of recruitment videos intended to attract potential staff to the company. Through a qualitative content analysis of these videos, I use the sociology of expectations and critical food studies approaches to edibility to demonstrate how complex and layered visions of desired futures for companies, communities, and individuals are intertwined into the performance of CM as both edible and producible as a novel but world changing desirable foodstuff. The notion of “producibility” is the key theoretical contribution of the paper, as an aligned concept to the existing work in edibility, that renders explicit the work of making new food technologies, and the identity work that operates in concert. I also make the case for the significance of recruitment videos as sites of symbolic work that are deserving of social science scrutiny.

Recruitment is an important issue for CM companies. The move from a small university-centered sector to a venture capital-backed start-up approach has shifted the culture and needs of the community (Stephens, Sexton, and Driessen 2019). The increasing importance of human resources in this context was made clear by Isha Datar, the Executive Director of New Harvest, the first nonprofit dedicated to promoting CM. In New Harvest’s 2018 annual report, she wrote “[f]ive years ago, I would say the limiting factor in [cultured meat] was funding. Today, I would say the limiting factor is skilled technical expertise” Datar (2018). Developing and attracting a skilled labor force has remained an ongoing activity for Memphis Meats and companies like it. But, I argue here, recruitment is more than just getting skilled people in the company. It is part of a much broader tapestry of activities that enact meanings and identities in the present and in the future for CM, those who eat it, and, importantly, those who produce it.

Before progressing, it is important to address an issue that has been key to the emergent field, but it not a core focus of this paper: the nomenclature used to describe the tissue. There has been ongoing plurality and debate over the terms used. The first wave of researchers in the 2000s typically used in vitro meat, then shifting toward cultured meat in the first half of the 2010s. Memphis Meats’ early promotional work used cultured meat. Then, in 2016, third sector meat-alternatives advocacy group the Good Food Institute were key to pushing the term clean meat, as a phrase that directed conversation to the positive aspects of why it was clean (as opposed to the potentially yuck-inducing aspects of why it was cultured), and that had scored well in consumer reaction tests. By 2017, the term cell-based meat has been promoted, including by Memphis Meats, in response to the weaknesses of clean, which angered the livestock industry for implying their meat was dirty, and seemed insufficiently technical to suit regulators. By late 2019, the Good Food Institute had started to back another new term, this time cultivated meat, and again various actors decided which of the multiple available names they should adopt. One key observation here, is that all of these terms retain the word “meat”. A central component of the CM community’s narrative in recent times is that their tissue is meat, just like meat from an animal, only produced in a different way.

### The sociology of cultured meat

CM has attracted a wide variety of social science analyses. These include publics or consumer-orientated research using focus groups (Verbeke et al. 2015; O’Keefe et al. 2016; Van Der Weele and Driessen 2019), experiments (Siegrist and Sütterlin 2017; Bekker et al. 2017) and surveys (Hocquette et al. 2015; Wilks and Phillips 2017, see Bryant and Barnett 2018 for a review) of peoples’ perceptions. Another recurrent theme has been ethical (Pluhar 2010; Schaefer and Savulescu 2014), legal (Petetin 2014; Johnson 2019), and political analysis (Driessen and Korthals 2012; Lee 2018) of the potentials for CM. There has also been a set of analyses of media and cultural representations of CM (Goodwin and Shoulders 2013; Laestadius and Caldwell 2015; Hopkins 2015), as well as those in fiction (McHugh 2010; Miller 2019).

This paper is situated within yet another stream social science research on CM that analyzes the community of researchers that advocates developing the technology. This typically emphasizes how the promises of CM are articulated and substantiated (Chiles 2013; Jönsson 2016; Stephens, King, and Lyall 2018), and how that contributes to rendering CM an ontologically stable and knowable entity (Stephens 2013; Sexton 2018; Mouat and Prince 2018). These authors typically stress the ambiguous status of CM and document the political drivers and practical mechanisms used to close this ambiguity. As these papers show, the dominant framing of CM as favored by the community that seeks to produce it is that it is meat, exactly the same as livestockbased meat, only made in a different way that minimizes environmental impact, animal use, and exposure to animal-borne disease or farm-used antibiotics. This framing clearly asserts CMs foodness and edibility while writing out any possibility that it is a meat alternative (such as the myriad other veggie burgers and sausages). This framing has been contested by some, most notably in recent time organizations in the US livestock industry, who brand it fake meat, or seek to prevent it from using the world meat at all (United States Cattlemens Association 2018; Johnson 2019).

### Theoretical context: expectations, edibility, and producibility

Explicitly or implicitly, this STS stream of writing on CM draws upon the Sociology of Expectations literature that has analyzed how visions of potential sociotechnical futures shape what happens today (Borup et al. 2006; Brown and Michael 2003). Examples of this work on other topics includes Berti and Levidow (2014) on biofuels, Morrison (2012) on regenerative medicine, and Lupton (2017) on 3D printed food. In a recent addition to this literature, Michael (2017) introduces the concepts of big futures and little futures. The former relates to globalized visions, such as the anthropocene or an age of migration. The latter relates to the mundane experiences and aspirations of daily life, which are also meaningful in peoples’ sense-making practices. He uses these concepts to explore the inter-relation of both that emerge as an ecology of futures, where sets of big and little futures can perform each other into being, or be set in competition and undermine their potential enactment.

I also draw upon another example of the Sociology of Expectations used in a biomedical context, work analyzing the practical experience of staff working at an anonymous biobank that closed over a period after the institutions that funded it declined to provide a second round of finance (Stephens and Dimond 2015i). The paper identifies three sub-categories of expectations – “field”, “institutional” and “individual” expectations – that describe the relationships between futures and presents that are understood and enacted at the level of a community, a single organization, and a single person. In the biobanking case, the field was human tissue biobanking, the institution was the specific biobank that closed, and the individuals were the staff that worked there. The story of the biobank that closed is one of a cycle of promise and disappointment. At first, the biobank articulated a future in which disease-specific tissue would be ethically stored long term and made available for research. Then, its core funding was removed and it faced potentially having to destroy its tissue collection. It then launched a bid to pass the material onto other biobanks, which had some success, although eventually the remaining funds ran out and it had to destroy the remaining samples (see also Stephens and Dimond 2015ii). The analysis describes the tug and pull between the projected futures for the field of biobanking, the specific biobank itself, and the staff who worked there as their expectations were repeated reconfigured, ultimately before the biobank was disbanded and the staff either left their jobs or we made redundant.

A second literature I draw upon in this paper is the emergent discussion by critical food scholars of “edibility” as a category and set of practices that are enacted in rendering a substance as food (Roe 2006; Abbots 2016). Roe (2006) draws our attention to the concept of “things becoming food” through meaning-making events, which, ultimately, are tied to eating. She argues that “edibility is a process, something that is performed, something enacted, and not something that necessarily demands rational, logical reasoning … things become food through how they are handled by humans, not by how they are described and named” (Roe 2006, 112). This literature includes multiple accounts of how certain materials become food, and in some instances then stop being food, including kangaroo meat (Waitt 2014), avocadoes (Charles 2002), organs (Wansink 2002), placenta (Vaughn 2019) and food waste (Blichfeldt, Mikkelsen, and Gram 2015; Waitt and Phillips 2016).

One part of this literature examines how animal bodies become edible as meat (Evans and Miele 2012; Morgan and Cole 2011; Stewart and Cole 2009; Vialles 1994). Another assesses how alternative proteins are framed as meat or meat-like (House 2018; Sexton 2016, 2018; Yates-Doerr 2015). In this line, Sexton (2018, 587) argues alternative protein developers, including those pursuing CM, “are engaged in multiple strategies that aim to materially and discursively construct the edibility of their products – that is, to convince consumers that their products are in fact ‘food’”. Roe (2006) and House (2018) highlight the multiple sets of actors engaged in performing edibility. Roe argues analysts should move beyond analyzing through the concepts of consumers and producers, to instead focus upon bodies, in her case the embodied practices of consumers. House captures this required multiplicity in arguing edibility is a network effect of diverse groups.

In this paper, I combine the sensibilities of both the sociology of expectations and these works on the performativity essential to edibility to argue that, through network effects, CM is positioned as edible and – the novel theoretical contribution of this paper – also “producible” through enactment of specially configured futures and presents at individual, institutional, field and community levels. This work is also embodied, as entangled as it is with the anticipation and conduct of labor in spaces of production, here the research and design laboratories and future pilot-plants. While many foods require some form of enactment as producible, this is particularly so of novel and boundary challenging foods that result from scientific labor, such as CM. The lack of existing practices and identity roles for production means these practices and identities need to be brought into being. Such world-making demands social science analysis. In the context of CM, as Iwill show, rendering this novel potential food as “producible” is akey parallel step in the symbolic and material production of embodied edibility.

## Methods

In this paper, I deploy these concepts of big and little futures, field, institutional and individual expectations, and edibility and producibility, to analyze the rising optimism of CM company Memphis Meats during a period of expansion. Specifically, this paper conducts a qualitative content analysis of their nine promotional films on YouTube, with a specific focus upon four videos produced by Memphis Meats after it achieved its 2017 Series A funding round of 17m USD to recruit new staff. The videos that have been analyzed are detailed in Table 1.

All videos were transcribed for text, visual and audio accompaniment. The transcripts were thematically coded for key narratives and categories of representation. I also recorded and analyzed the job adverts on Memphis Meats website at three points over a year, on December 10th 2018, June 5th 2019, and December 10th 2019. The analysis is informed by my sustained research engagement with the CM field since 2008, work that has involved over 50 interviews with actors in the field, attendance at most major events during this period, and ongoing analysis of the narrative framing of CM circulated by those developing it. The broader project has been approved by Cardiff University and Brunel University London research ethics committees.

Memphis Meat’s recruitment videos are important to analyze because they account for a different type of messaging to the type analyzed elsewhere. Studies of public messaging around CM have focused upon narratives with an assumed audience of potential consumers, funders, and to a limited extent regulatory bodies (Dilworth and McGregor 2015; Sexton, Garnett, and Lorimer 2019; Van der Weele and Driessen 2013). These recruitment videos, in contrast, offer insight into messaging directed at potential colleagues and insiders within the CM community. Furthermore, they are important as symbolic representations of the significance of recruitment to the current start-up sector, and values, skills, and attitudes sought by the company. Subsequently, this paper contributes by highlighting the significance of recruitment videos as sites for social science analysis, as an empirical setting that has until now been under-researched (with the exception of a set of papers on recruitment videos used by terrorist groups (Salem, Reid, and Chen 2008; Carriere and Blackman 2016; Leander 2017; Macnair and Frank 2017)).

### Memphis Meats

Memphis Meats were co-founded by Chief Executive Officer (CEO) Uma Valeti and Chief Scientific Officer (CSO) Nicholas Genovese. Valeti was a practicing cardiologist who had long held interests in the technology. Genovese had been among the first wave of researchers working on CM, having been based at the University of Missouri. They launched the company in 2015 and enrolled into the IndieBio program, a San Francisco-based biotech accelerator that provided a package for all enrolees including mentorship, laboratory space, and 200,000 USD of early-stage funding in exchange for an equity stake in the company. During their time in the program they produced a slick video featuring the revealing of the world’s first cultured meatball (Memphis Meats 2016), which was taste-tested and used as part of their strategy to leverage further funds at IndieBio’s “demo day” pitch event to investors. Following this, Memphis Meats successfully raised 2,750,000 USD in March 2016 through their seed round. Fourteen months later they released another video, this time showing cultured chicken and duck products (Memphis Meats 2017), and subsequently raised a further 17m USD during their 2017 Series A investment round. Both of these videos will be analyzed in the following section. The company attracted a range of investment partners, including mission-based venture capitalists such as New Crop Capital, celebrity investors including Bill Gates and Richard Branson, and strategic investments from meat producers Cargill. In January 2020, Memphis Meats announced they had closed their Series B funding round, raising 161m USD of investment, with the major investors being SoftBank group, Temasek, and Norwest. With this, they also announced plans for their first pilot-plant for making CM, and their intention to expand their staff numbers from around 40 to around 130, situating them as one of the leading CM companies in the world (Purdy 2020). This given, Memphis Meats are still operating in the research and design stage, and are yet to release any products.

At the time of writing, Memphis Meats employs around 40 people. Across late 2018 and 2019, they advertised a number of posts, ranging between four and nine potential appointments at the three survey points. A number of the job titles would not look out of place in a biomedical tissue engineering laboratory, including roles such as Senior Research Associate: Cell Line Development, Director of Tissue and Bioprocess Engineering, or Senior Data Scientist. Other roles, such as Meat Product Technologist and Product Formulation and Performance Lead, speak more explicitly to food development. Frequently each job advert includes the same “About Us” text, noting:

“At the forefront of farming and food, Memphis Meats is innovating new ways to produce real meat, without the need to breed, feed and slaughter animals. Our process begins with an animal cell, which is cultivated and harvested as meat. Memphis Meats is a mission-driven company focused on bringing to meat lovers an authentic product that is truly humane, sustainably produced, nutritious and delicious! We are now expanding our team of dedicated professionals across disciplines to make better meat for a better world.” (Memphis Meats 2019)

This “About Us” text foreshadows themes found in the recruitment videos, of asserting the status of the tissue, its moral desirability, and transformative capacity. In what follows, I will explore these issues in more depth, first by analyzing Memphis Meats’ prototype demonstration videos, and then the four key recruitment videos release in 2017.

### “The meatball that changed the world”: promotional narratives in Memphis Meats’ prototype demonstration videos

Memphis Meats posted their first YouTube video in January 2016, as they came to the end of their IndieBio accelerator program. It features CEO Uma Valeti describing his excitement that the company had produced a CM meatball, over a montage of scenes showing the meatball being prepared, cooked, and tasted by an unnamed woman. In it, Valeti explains: “we started Memphis Meats to make eating meat delicious, safe and healthy for everyone. We have had this technical breakthrough of using real meat cells to grow meat”. Cutting to Valeti posing with the meatball, he smiles as he declares it “the meatball that changed the world”. The narration continues, “There are not enough resources to feed a growing world population. The greenhouse gas from our process is 90% less than what traditional animal agriculture would cause.” He explains the team have been working on producing a meatball over several weeks, and introduces chef Dave Anderson who is shown cooking it. Valeti then invokes the sensuality of food as he tells how Anderson “took the meat that we grew, and he used a recipe – an Italian meatball recipe – and created a beautiful meatball, and we watched him cook it, we watched how the meatball reacted when it was put in a pan, we heard the sizzle, we smelt the meat that was cooked, and it as exactly what you would expect a beef meatball to smell like”. At this point an unnamed woman takes a bite of the prepared meatball, that has been ascetically positioned on pasta with olives, as she declares “it tastes like a meatball, its good”. In closing, Valeti summarizes “you know this is the first time a meatball has ever been cooked with beef cells that did not require a cow to be slaughtered, so this is a big and momentous occasion for us, and given that it tasted exactly like beef, it is one of the historic moments in the history of our company, and I am glad you are here to capture it”.

Just over a year later, in March 2017, Memphis Meats published a second major tastetest YouTube video. Again, Valeti narrates “Hi, Uma Valleti here from San Francisco. One year after making history with the world’s first clean meatball, we are at it again. I’m proud to unveil the world’s first ever poultry products that were grown from animal cells without the full animal. Today we are unveiling two types of meat. Chicken and duck.” As the video progresses, Valeti again provides an account for why CM is needed, “With the demand for meat growing so quickly there are not enough resources to feed the planet. We need to completely change the way meat gets to the plate”. An invited taster is then shown eating the CM, as he happily announces “It tastes like chicken, Yes!”. Valeti continues, “We believe this to be one of the biggest technological leaps for humanity. Our goal is to make delicious clean meat that is better for you, the environment, and the animals, and most importantly better for your favorite recipes. Today we have taken an historic step toward this goal of a better meat and a better world.”

Both videos weave together big and little futures, as they assert both the positive transformation CM could deliver, and the day-to-day but joyous sensuality of cooking and consuming food. Neither video heavily details any potentially catastrophic impact of conventional meat production on the environment or animal welfare – there is no sense in which the videos seek to promote guilt or regret in the viewer for conventional meat consumption – while still allowing those big, dangerous, futures to feature. Foodness is asserted repeatedly across both videos, with discussion of an Italian meatball recipe, sizzle and smell, esthetically considered visuals of CM attractively displayed on plates, and happy eaters taking their first bites. As such, the videos work to situate the CM prototypes as familiar and desirable – edible – while also transformational, and render what is innovative and unprecedented as somehow normal, a communing of big and little futures tied together in a delicious social good. The promise here is of consuming great tasting and world-changing meat products, but a meat product that has the capacity to redefine how meat is made and understood.

This address, and these invocations, are common in CM promotional materials, and are typical of the interlacing of promise and ontology as the status and benefits of CM are asserted, here through video. This point, however, has been made elsewhere (Jönsson 2016; O’Riordan, Fotopoulou, and Stephens 2017; Sexton 2018). The next section analyzes a much less common address within the community; that orientated toward potential future employees. I now analyze this set of four films in detail.

### “How many people literally get to invent the future and turn it into reality?”: promise and producibility in Memphis Meats’ recruitment videos

Memphis Meats have released four recruitment videos on YouTube, the first on August 22nd, 2017 and the following three a month later on September 26th. The first contrasts to the latter three in that it has no vocal narration, instead accompanied by lively and progressively anthemic folk rock instrumental music. This given, all four videos seem to be part of a single campaign as all four feature shared references and the featured staff wear the same clothes, suggesting they were filmed on the same day. We discuss each in turn.

#### Video one: Join our team. Change the world

Starting by asking “What is Memphis Meats?” the video shows a staff member greeting other smiling team members in the Memphis Meats office. The video subsequently cuts between shots of staff in the work place with images of Memphis Meats’ achievements to date and pictures related to its mission. Over the course of its duration the words “Discovery”, “Mission”, “Creativity”, “Challenge”, “Vision”, “Growth” and “Community” are shown over images of office work, team photos, idealized farm animals, beautiful mountains, cell culture work, and the Memphis Meats team collectively engaging in board meetings, nacho consumption, discussions around microscopes and white boards, table tennis, board games, playing with a dog, and group exercises. This is inter-dispersed with footage from Memphis Meats’ two videos that revealed the cultured meatball and poultry dishes. Other text includes shots of newspaper stories, such as Fortune Magazine’s “the hottest tech in silicon valley”, a framed Wall Street Journal story titled “Quest heats up for alternatives to beef”, and another stating “Startup serves chicken from the lab”. We are also shown Memphis Meats’ staff presenting PowerPoint slides declaring “We are the clear leaders of the clean meat movement. We have significantly de-risked clean meat production in two years” and “Meat is an enormous market opportunity”, suggesting a “$750b” global figure. The video closes with the team using scrabble pieces to spell “Memphis Meats”, before the final shots that close all four videos, the slogan “Join our team. Change the world” and a link to their careers webpage.

#### Video two: vision at Memphis Meats

The second video is the first to use a format that is standard across the three released in September 2017. It features Memphis Meats staff members speaking directly to camera, typically sat at a desk just meters away from the lens, describing life in the company and what is most likely, at least in part, a semi-planned account of why working there is aspirational. Speakers and their job titles are written onscreen as each speaks the first time. It begins with Eric, a senior scientist, who asks “how many people literally get to invent the future and turn it into reality?” This opening line sets a tone of epic scale and opportunity associated with working at the company. It explicitly asserts the transformational capacity of the CM by linking a potential desirable future to the day-to-day activity of employment at Memphis Meats. Eric’s confident delivery, inspirational tone, and stylish-come-hipster clothes contrast to next speaker, research scientist Michaela, whose less outlandish clothing and less polished delivery provide a more every day and human tone to what remains an aspirational and transformation message: “working at Memphis Meats is a challenge every day in the best possible way. You are working on the cutting edge of science and technology in a way that is going to make the world a better place, and that’s a great feeling.” Following this, formulation scientist Morgan and cultivation engineer Matthew stress the need for creativity and invention on the job. Then, CSO & Co-founder Nick provides the first of three consecutive statements on the need for multiple skillsets: “in order to make this work, to bring this to the public, this is going to take expertise across biology, across chemistry, across food technology, across automations science, and public relations and outreach”. CEO & Co-founder Uma then comments that they need “people with love for food, love for culinary creativity, as well as using science and innovation.” The final statement is a second appearance for Eric, who confidently, looking straight into camera, says “what’s really funny is that as a scientist my job starts from not knowing something, the unknown, and at Memphis Meats we start from there. We ask a question we don’t know the answer, but we quickly figure it out, apply it through engineering, and turn that into a food product. That’s what I love about the science here. Fast, efficient and delicious.”

#### Video three: mission at Memphis Meats

The third video starts with a voice over a black screen, that we soon find belongs to Steve, VP of business development. We see his smiling face, as he declares “this is the first thing I’ve done where I just can’t stop talking about it. Morning day and night. I wake up and I talk about it, I go home and I talk about it, I tell my wife about it, I tell my friends about it all weekend long”. Next, speaking more calmly, but with more assurance, Head of Mission David, explains “I think it’s incredibly motivating to know that what you are doing is going to impact the world in an incredibly positive way”, a sentiment echoed by research scientist and lab manager Danielle who says “you are working for a team that is going to change the world”. Returning to David, the viewer is offered an account of the diverse motivations of those working at Memphis Meats: “some folks are interested in it from the environmental perspective, some people care about animal welfare, some people care about access to food, and food health and safety”. Matthew is then the first of three speakers to express their personal enjoyment and motivation at work, “one of my personal goals is to make the most amazing and most delicious piece of meat that’s ever been made.” After these, David again describes the diversity, here introducing the inclusive sentiment among staff: “we have vegans and vegetarians, and we have diehard meat eaters, and everything in between, and everyone is welcome because everyone is united by the same common goal of making clean meat a reality”. CSO & Co-founder Nick articulates more fully the logic behind the company’s mission: “If we can produce meat from animals’ cells and not the animal itself we can save resources required to produce our food”, before Eric again charismatically and enthusiastically closes the interviews stating “If you love food, if you love science, if you love engineering, and if you love mission-based work, and you get to put all of that all together and feed the world, Memphis Meats is the place to do it”.

#### Video four: culture at Memphis Meats

The fourth and final video begins with research scientist Michaela, who exudes a subtle warmth and happiness as she explains, “waking up every morning and knowing I get to work at Memphis Meats is one of the greatest things about waking up each morning”. David and then Danielle again stress the diversity of staff expertise and motivations, before David reasserts “but everybody’s kind of united by one common goal to make clean meat happen”. This video then takes a different focus to those before it, stressing the community and social nature of working in the company. Michaela notes people have “great senses of humor”, Matthew explains “I can always count on my team mates to be there, they’ll bring me dinner sometimes when I’m late”, and Steve suggests “the folks are amazing, and at the same time there are no egos at all”. Danielle explains “We have a variety of extra-curricular activities … anything from hiking to watching movies together”, while Morgan adds “a lot of board game nights, go get beers after work” to the list. CEO & Co-founder Uma then says “anyone who wants to join this team, who has the right sense of purpose, and wants to be among a creative group of people who are collaborating and brain storming to solve problems would find this as a natural home” before Danielle asserts “you are working for a team that is going to change the world”, and Michaela closes the interviews with “it’s a great team, it’s a great place, it’s a great challenge, I’m happy here”.

## Discussion

Memphis Meat’s recruitment videos exhibit a set of key themes that collectively work as an invitation to potential employees into their world by aligning both big and little futures, as well as individual, institutional, and field expectations. Little futures in the company are invoked through references to collegiality, friendship, and the day-to-day mutual support of a helpful work environment, particularly in video four, that describes how colleagues “bring me dinner”, doing “extra-curricular activities” with “great senses of humor”. This, we are told, occurs within a diverse environment of people with different expertise and different motivations. It is here that the individual and institutional expectations are weaved into alignment as this group of diverse, happy people, focus upon a common goal. This shared aim is rendered highly desirable morally in terms of animal and environmental safeguarding, and both aspirational and achievable, through the immediate and little future of interdisciplinary work at the cutting edge of scientific research. Individual expectations, and the personal, are introduced outside of work through references to family, friends, and extra-circular activities. They are also articulated within the work environment through references to personal work-based achievements, and personal and company “mission” compatibility. Through this, the little and big futures are narratively aligned, as this innovative and driven collective are repeatedly situated upon a frontier of knowledge and food-system change. This dynamic is captured cleanly in Eric’s question to camera – “how many people literately get to invent the future and turn it into reality” – as well as the video set’s recurrent closing theme, “Join our team, change the world”. The direct link between global transformation and day-to-day employment is rendered clear and explicit.

While these links of big and little futures, and individual and institutional expectations, are writ front and center of the narrative, the connections between Memphis Meats as an institution and CM production as a field are more subtle. In this company-specific narrative, the broader field is both hardly mentioned and also subsumed within the story of the company’s own mission and success to date. The only explicit reference to a wider CM community features in the slide projected on the wall describing Memphis Meats as “clear leaders of the clean meat movement”. The narrative seeks to situate the company both at the frontier of technology, and their own community. This same slide also makes a rare reference to an economic potential for the institution and field, as an “enormous market opportunity”. This given, far more frequently stressed, is the transformative moral and environmental good of the technology that is presented as the unifying cause. Through this messaging, big and little futures, and individual, institutional and field expectations, are intertwined and co-enacted through representations of the positive emotional experience of collective work, positive change, frontier activity, and moral significance.

However, these recruitment videos do further symbolic work. Like the earlier videos premiering new demonstration cultured meatballs and poultry products, these recruitment videos also seek to assert the status of CM itself, rendering it as food, real, and delicious. All the videos invoke the notion that CM is food that is both knowable and familiar, while also transformative and world-saving. This theme is evident in the recruitment videos in CEO Uma’s statement that Memphis Meats need “people with love for food”, and Eric’s declaration that their science remains “fast, efficient and delicious”. As Sexton (2018, 587) argues, this works to “convince consumers that their products are in fact ‘food’”.

But more than this, the recruitment videos work to assert foodness also to potential employees and future producers of CM. It works to position CM as both edible and “producible”, a knowable potential accomplishment within a multi-layered desirability, as delicious, as collegiate, and as morally significant. As such, the videos invoke, offer, and render desirable a novel identity category: the CM producer. The narrative articulates a vision for the first employees of a nascent potential industry, as well as a new trajectory for food production practices. The production of this identity category as a CM producer, and the attendant process of producibility, is essential to stabilizing a notion of what CM is and what its future may hold. It seeks to define who will make it, how they will understand themselves, and why.

This given, the CM producer is also an inherently promissory character, premised upon anticipated future production of CM resultant from the paid labor of R&D to be pursued in the near future. As such, as Roe (2006) argues of edibility, producibility is premised upon (actual and anticipated) embodied action – and collective embodied action – of Memphis Meats’ employees engaging in the shared labor of physically and symbolically bringing CM into being. This enactment of an identity category, and its furnishing with an assumed ethics, is essential for the instantiation of an emergent industrial sector.

As such, these videos have a role in what I term the ‘process of becoming’ CM is currently undergoing, a small step among wider processes that render it edible *and* producible. Within the micro-ecology of Memphis Meats recruitment videos, the big futures of world-changing frontier food science, and the little futures of a supportive, happy, but research-active work environment all co-enact collectively. By design, these videos literally work to bring the positive CM big futures into being by seeking to enroll potential employees into the little futures of paid work on making and promoting CM. They are symbolic projections with the capacity to materially effect this emergence technology sector by defining its agenda and attracting people to conduct the associated labor.

This given, these videos alone are, of course, insufficient to perform such social change unsupported. Instead, they operate as part of a much broader network effect (House 2018) that includes among other elements the culture of venture capital investment, representations in mainstream media, and the materiality of laboratory research and design itself, all of which contribute to the producibility of CM. Such producibility is material and symbolic, as a multifaceted socio-technical assemblage in a state of emergence, working toward a world in which CM is both regularly eaten, and delivers upon its proposed moral gains.

So, given this intentionality, it remains important to consider House’s (2018) observation that edibility and consumption are *not the same thing* – it is possible for a food to be positioned as “edible” without it regularly being eaten. This is also true of consumption and producibility. Just because something becomes producible, it does not follow it will necessarily be produced and consumed. This is particularly so as the big futures of CM have already attracted a set of counter futures articulated by groups such as the U.S. Cattlemen’s Association (2018), European Livestock Voice (2020), and Clean Meat Hoax (2020) who try to undermine these narratives in a range of ways for a range of purposes. Such ecologies of the future also demand critical scrutiny, as is their role in the unfolding and indeterminate process of CM’s becoming.

In this paper, I have developed the notion of producibility, as an allied concept to that of edibility, that is necessary for analyzing novel food technologies such as CM. I have demonstrated the enactment of producibility in the recruitment videos of the first CM start-up company Memphis Meats. Here both edibility and producibility are co-enacted as the symbolic proposing of CM as food is entangled with the (paid) labor of its production. This engenders a novel professional identity – the CM producer – and asserts an aligned moral disposition. As such, I have demonstrated that recruitment videos are important sites for social science analysis as a distinct and underassessed cultural form.

As noted, CM remains in a process of becoming, a process in part framed by the intentionality of the community who seek to produce it. This is a becoming of the food itself, as knowable and edible, but also the becoming of the individuals, communities, and practices entwined within its production. Their identities, ambitions, and futures – as individuals, institutions, and fields – are in part enacted by these recruitment videos, and remain core to the potential unfolding of CM futures big and little, whatever form they may take.

## Figures and Tables

**Table 1:** Analyzed videos for Memphis Meals.

Promotional video title/URL	Duration	Publication date
The World’s First Cell-based Meatball – Memphis Meats https://www.youtube.com/watch?v=Y027yLT2QY0	1.32 m	31/1/16
First Cell-based Meat Taste Test – Memphis Meats https://www.youtube.com/watch?v=FXpDiZUevPY	0.40 m	16/2/16
World’s First Clean Beef Fajita – Memphis Meats https://www.youtube.com/watch?v=3nCickhu3xI	0.15 m	22/11/16
Meet Team Meat! https://www.youtube.com/watch?v=-SgftNDd5QY	0.48 m	22/11/16
Historic first: cell-based poultry tasting https://www.youtube.com/watch?v=b5ezRx23EMg	1.31 m	21/3/17
Recruitment video title/URL	Duration	Publication date
“Join Our Team, Change the World” https://www.youtube.com/watch?v=hCr-ZoDgxdI	1.24 m	22/8/17
“Vision at Memphis Meats” https://www.youtube.com/watch?v=S7rBwk673b4	1.43 m	26/9/17
“Mission at Memphis Meats” https://www.youtube.com/watch?v=4Waa-HTPwjY	1.37 m	26/9/17
“Culture at Memphis Meats” https://www.youtube.com/watch?v=LltWsfDAkN0	1.43 m	26/9/17

All promotional videos accessed 5/6/19.

All recruitment videos accessed 7/11/18.

video titles can change on YouTube, titles listed here as on access date.
